# Pan-caspase inhibitor protects against noise-induced hearing loss in a rodent model

**DOI:** 10.3389/fnins.2025.1497773

**Published:** 2025-02-10

**Authors:** Maria Fernanda Yepes, Kayla Minesinger, Federica M. Raciti, Maria Camila Salazar, Suhrud M. Rajguru

**Affiliations:** ^1^Department of Neuroscience, University of Miami Miller School of Medicine, Miami, FL, United States; ^2^Department of Biomedical Engineering, University of Miami, Coral Gables, FL, United States; ^3^Department of Otolaryngology, University of Miami Miller School of Medicine, Miami, FL, United States

**Keywords:** NIHL, noise induced hearing loss, cochlea, ABR, auditory brainstem responses, inner ear therapeutics, caspase inhibitor

## Abstract

**Background:**

Despite the high prevalence of noise-induced hearing loss (NIHL), no effective treatments exist currently. Underlying mechanisms behind NIHL include elevated reactive oxygen species and inflammation, all which ultimately lead to cellular apoptosis. Z-VAD-FMK, an apoptosis inhibitor, has demonstrated protective effects against cochlear hair cells exposed to ototoxic agents; however, its potential for treating NIHL remains unexplored. This study assessed the efficacy of Z-VAD-FMK as a therapeutic for noise-induced cochlear injury in a rodent model.

**Methods:**

Rodents were assigned to one of four groups: (1) unexposed, (2) noise-exposed, (3) noise + vehicle, and (4) noise + Z-VAD-FMK. Noise delivery consisted of 1 h of 110 dB continuous white-noise, with Z-VAD-FMK administered intraperitoneally 6 h afterward. Auditory brainstem responses (ABRs), cochlear hair cell density, and protein levels were evaluated post-interventions.

**Results:**

Noise exposure caused a permanent threshold shift across all frequencies, with minimal recovery by day 28. However, post-exposure treatment with Z-VAD-FMK significantly mitigated ABR threshold, amplitudes, and latencies shifts particularly at low and mid frequencies. Treatment rescued outer hair cells across middle and basal cochlear turns and reduced caspase-9 and IL-1β levels, as indicated by protein analysis.

**Conclusion:**

Our findings indicate that a single intraperitoneal injection of Z-VAD-FMK can partially mitigate cochlear dysfunction induced by acoustic overexposure in a rodent model, highlighting its potential as a therapeutic intervention for NIHL.

## 1 Introduction

Noise-induced hearing loss (NIHL) is an irreversible condition affecting millions worldwide, with significant implications for quality of life and public health (Stucken and Hong, 2014; Basner et al., 2014; Arlinger, 2003). Despite advances in hearing protection and awareness, the lack of effective pharmacological interventions leaves individuals vulnerable to the detrimental effects of acoustic trauma. The pathophysiology of NIHL is complex and multifaceted, involving a cascade of damaging events within the cochlea that includes oxidative stress, free radical production, neuronal excitotoxicity, and the generation of reactive oxygen species (ROS) (Ohinata et al., 2000; Ohlemiller et al., 1999; Van Campen et al., 2002; Puel et al., 1998; Pujol et al., 1993; Zhao et al., 1996; Hu et al., 2002; Le et al., 2017). These processes collectively disrupt neuronal integrity and compromise hair cell survival by activating programmed cell death pathways, such as apoptosis, as indicated by elevated levels of caspases 3, 8, 9, and 10 in inner and outer hair cells following noise exposure (Hu et al., 2002; Hu et al., 2006; Nicotera et al., 2003; Pirvola et al., 2000; Gregoli and Bondurant, 1999). The resulting molecular and cellular damage leads to functional deficits, including elevated auditory thresholds and diminished auditory brainstem response (ABR) wave amplitudes (Kujawa and Liberman, 2009).

Currently, no FDA-approved treatments exist to prevent or alleviate NIHL, with existing strategies focusing primarily on ear protection and limiting noise exposure. Although preclinical research has explored various therapeutic agents—such as antioxidants (Kopke et al., 2000; Lu et al., 2014), lipid peroxidation inhibitors (Ohinata et al., 2000; Quirk et al., 1994), vasodilators (Ceylan et al., 2019; Kansu et al., 2011; Sakat et al., 2016; Davidenko and Antzelevitch, 1984), vitamins (Sies and Stahl, 1995; McFadden et al., 2005), and calcium modulators (Shen et al., 2007)—these approaches have yet to be successfully translated into clinical practice, likely due to conflicting results, challenges in drug delivery, and potential side effects. Consequently, the search for new and effective therapeutic interventions remains a pressing priority.

One promising approach involves the use of Z-VAD-FMK, a pan-caspase inhibitor derived from benzyloxy carbonyl and fluoromethylketone (Chauvier et al., 2007). Z-VAD-FMK functions by binding to the catalytic site of caspases, effectively halting both the initiation and execution phases of apoptosis (Gregoli and Bondurant, 1999; Chauvier et al., 2007). Its mechanism of action has been visually corroborated by electron microscopy, which shows a reduction in apoptotic features in cultured cells treated with Z-VAD-FMK (Salvesen and Dixit, 1997). Initially explored as a protective agent in cardiac ischemic injuries (Huang et al., 2000; Iwata et al., 2002), Z-VAD-FMK has since demonstrated efficacy in auditory research, particularly in preventing cochlear hair cell loss induced by ototoxic agents such as gentamicin, actinomycin-D, and streptomycin (Chang et al., 2020; Matsui et al., 2003; Cheng et al., 2003). Notably, Z-VAD-FMK’s broad inhibition of multiple caspases offers superior protection for cochlear hair cells compared to therapies targeting individual caspases (Cunningham et al., 2002). *In vivo* studies further reinforce these findings, with rodents treated with Z-VAD-FMK showing significantly lower ABR threshold shifts after exposure to ototoxic agents compared to untreated controls (Okuda et al., 2005). So far, there is only one study that has explored the potential of Z-VAD-FMK in protecting against noise-induced cochlear damage, demonstrating reduced ABR threshold shifts in rodents subjected to a single impulse gunshot trauma (Abaamrane et al., 2011).

In this study, we investigated functional, histological, and protein-level changes to elucidate the neuroprotective effects of Z-VAD-FMK on the auditory organ following continuous noise exposure.

## 2 Methods

### 2.1 Experimental design

The use of rodents in this study was approved by the University of Miami Animal Care and Use Committee and adhered to the NIH Guidelines for the Care and Use of Laboratory Animals. Equal number of male and female Brown Norway rats (15–17 weeks old, Charles River Laboratories) were randomly allocated into four groups: (1) unexposed (*n* = 8), (2) noise-exposed (*n* = 8), (3) noise + vehicle (*n* = 8), and (4) noise + Z-VAD-FMK (3 mg/kg) (*n* = 8). Functional assessments were conducted prior to any trauma or treatment, and subsequently at days 1, 3, 7, 14, and 28 post interventions. Euthanasia of all animals took place on day 28 for immunobiological studies. Protein quantification analyses were conducted on tissues 24 h after interventions.

### 2.2 Noise exposure

Acoustic exposure (4–8 kHz octave-band noise, 110 dBA, 1 h) was administered continuously to all animals. Rats were kept awake and unrestrained in equally spaced compartments within a cage, with one animal per compartment and four animals housed simultaneously. The cage was positioned above a rotating platform moving at a consistent speed. A soundproof chamber equipped with four speakers, evenly distributed, ensured uniform sound distribution. Prior to noise exposure, noise calibration was conducted using a Bluetooth SPL meter (Uni-T, UT353 Mini Sound Level Meter) placed inside the compartments. Sound pressure levels showed minimal variation (<2 dB) across different compartments within the cage. Animals were closely monitored at 15-min intervals to ensure their well-being throughout the procedure.

### 2.3 Drug delivery

Z-VAD-FMK (TOCRIS R&D Systems, Minneapolis, MN; Cat #2163), a broad-spectrum caspase inhibitor, was diluted in 10% DMSO and administered intraperitoneally at a dosage of 3 mg/kg. A single injection was given 6 h after the conclusion of noise exposure. Z-VAD-FMK was stored at −20°C and prepared immediately before each use.

### 2.4 Functional measurements

#### 2.4.1 Auditory brainstem response

Auditory brainstem responses (ABRs) were recorded both before and at days 1, 3, 7, 14, and 28 post-interventions. Animals were anesthetized using a cocktail of ketamine (44 mg/kg, i.m.) and xylazine (5 mg/kg, i.m), with supplemental half-dose injections administered as needed to maintain anesthesia. Body temperature was maintained near 37°C using a heating pad. Subdermal needle electrodes were positioned on the vertex, near the left leg, and ventrolateral to the left and right auricles. ABR recordings were conducted in a soundproof chamber using a pre-amplified data acquisition system (Intelligent Hearing Systems, HIS, Miami, FL). Stimuli were delivered at frequencies of 2 kHz, 4 kHz, 8 kHz, 16 kHz, 24 kHz, and 32 kHz, and response waves were recorded starting at 90 dB in decreasing 5 dB steps. Auditory sensitivity thresholds were determined as the lowest intensity at which response peaks, particularly wave I and IV, were visually detected. To assess the effects of trauma and treatment on the peripheral portion of the cochlear nerve, peak amplitudes and latencies of ABR wave I at a suprathreshold level of 80 dB were documented using a node-to-peak measurement. Threshold, amplitude, and latency shifts were calculated as the difference between post-exposure values to pre-exposure measurements. Data was reviewed by principal investigators and a blinded research technician for accuracy.

### 2.5 Immunohistochemistry and imaging

Twenty-eight days post-interventions, female and male subjects were euthanized via a cardio-perfusion technique. Cochleae were harvested and fixed with 4% paraformaldehyde (Sigma-Aldrich Inc., Missouri, #158127) for 2 days. Samples were subsequently washed three times with phosphate-buffered saline (PBS) and decalcified for 3 weeks using 10% ethylenediaminetetraacetic acid (EDTA, Sigma-Aldrich Inc., Missouri, #EDS). Specimens were stored in PBS with 0.05% Sodium Azide (Sigma-Aldrich Inc., Missouri, #71289) at 4°C until dissections were performed. For immunostaining, cochleae were incubated in a lysis buffer solution consisting of 0.3% Triton-X diluted in 1X PBS for 1 h. After three washes at 10-min intervals, samples were placed in a blocking solution of 5% normal horse serum diluted in 1X PBS. Alexa Fluor 546 Phalloidin (1:2,000, Invitrogen Cat #A2228), was then added to each sample for 1 h, followed by three 5-min washes before mounting on top of a 20uL Vectashield Antifade Mounting Medium with DAPI (Vector Laboratories Inc., Cat #H-1200) solution.

Cochleae were imaged using a High-Speed Confocal Microscope (Oxford Instruments, model 200) with excitation wavelengths of 546 nm for Phalloidin and 405 nm for DAPI. Confocal z-stack images of the apical, middle, and basal regions of the cochlea were acquired at 20× magnification. Cell counts of inner hair cells (IHCs) and outer hair cells (OHCs) were determined using ImageJ software.

### 2.6 Protein quantification

Cochlear tissues from male Brown Norway subjects were collected at 24 h after interventions and were immediately snap-frozen in liquid nitrogen. Total protein was extracted using ice-cold RIPA lysis buffer (Millipore Sigma, Cat #R0278) supplemented with a 1X protease inhibitor cocktail (Millipore Sigma, Cat #109M4068V). Samples were then homogenized for 15 s utilizing a handheld homogenizer fitted with a stainless-steel saw teeth bottom generator (Fisherbrand^™^ Model). Homogenates were then centrifuged at 12,000 g for 8 min at 4°C, after which the supernatant was collected. Protein concentration was measured using the Bradford Assay at a wavelength of 495 nm protein assay (ThermoFisher Scientific, Cat #1861426). Subsequently, 5X Laemmli loading buffer was added to the samples, followed by heating at 100°C for 6 min to denature the proteins. A 15 μL aliquot of each sample was then loaded onto an SDS-PAGE gel (Bio-Rad Laboratories, Cat #5678094) for electrophoresis, which was conducted for 1 h. Following electrophoresis, proteins were transferred to a nitrocellulose membrane (Bio-Rad Laboratories, Cat #1704157). For protein detection, primary antibodies were used as follows: Rabbit anti-Caspase-8 (1:1,000, Novus, Cat #NB100-56116) Rabbit anti-Caspase-9 (1:100, Santacruz Biotech, Cat #SC-7885), Mouse anti-IL-1β (1:1,000, Cell Signaling, Cat #12242S) and Rabbit anti-TNF-α (1:500, Invitrogen, Cat #PA1-4028). Membranes were incubated with the primary antibodies overnight at 4°C on a rocking platform. Following primary antibody incubation, membranes were washed with TBST for a total of 30 min and incubated with secondary antibodies, including goat anti-rabbit IgG (1:1,000, Cell Signaling Cat #7074S) and goat anti-mouse IgG (1:1,000, Cell Signaling, Cat #7076S), diluted in 10 mL of blocking solution at room temperature for 1 h. After secondary antibody incubation, the membranes were treated with ECL luminescence reagent (Cell Signaling Technology, Cat #95538S) for 1 min and developed using a Bio-Rad ChemiDoc MP Imaging system (Bio-Rad Laboratories, Cat #17001402). Vinculin (1:100, Invitrogen, Cat #14-9777-82) was employed as the internal normalization loading control. Bands were identified by accurate molecular weight estimation using Precision Plus Protein Dual Color Standards (Bio-Rad, Cat #1610374) and subsequently quantified using Image Lab 6.1 software.

### 2.7 Statistical analysis

Statistical analyses were performed to assess the significance of differences in mean and standard error (SE) values for ABR thresholds shifts, wave I amplitudes and latencies shifts, hair cell counts, and protein levels across different groups, frequencies, and time points. Statistical significance was defined as a *p*-value of <0.05. A mixed-effects model based on two-way ANOVA was employed to compare group means at each timepoint and frequency. For data that did not meet the assumptions of normality, nonparametric tests were applied. *Post hoc* Tukey’s multiple comparison tests and appropriate corrections were conducted to identify significant statistical relationships. All statistical analyses were performed using GraphPad Prism10.

## 3 Results

### 3.1 Post-noise delivery of Z-VAD-FMK significantly reduced auditory threshold shifts

**Figure 1:**
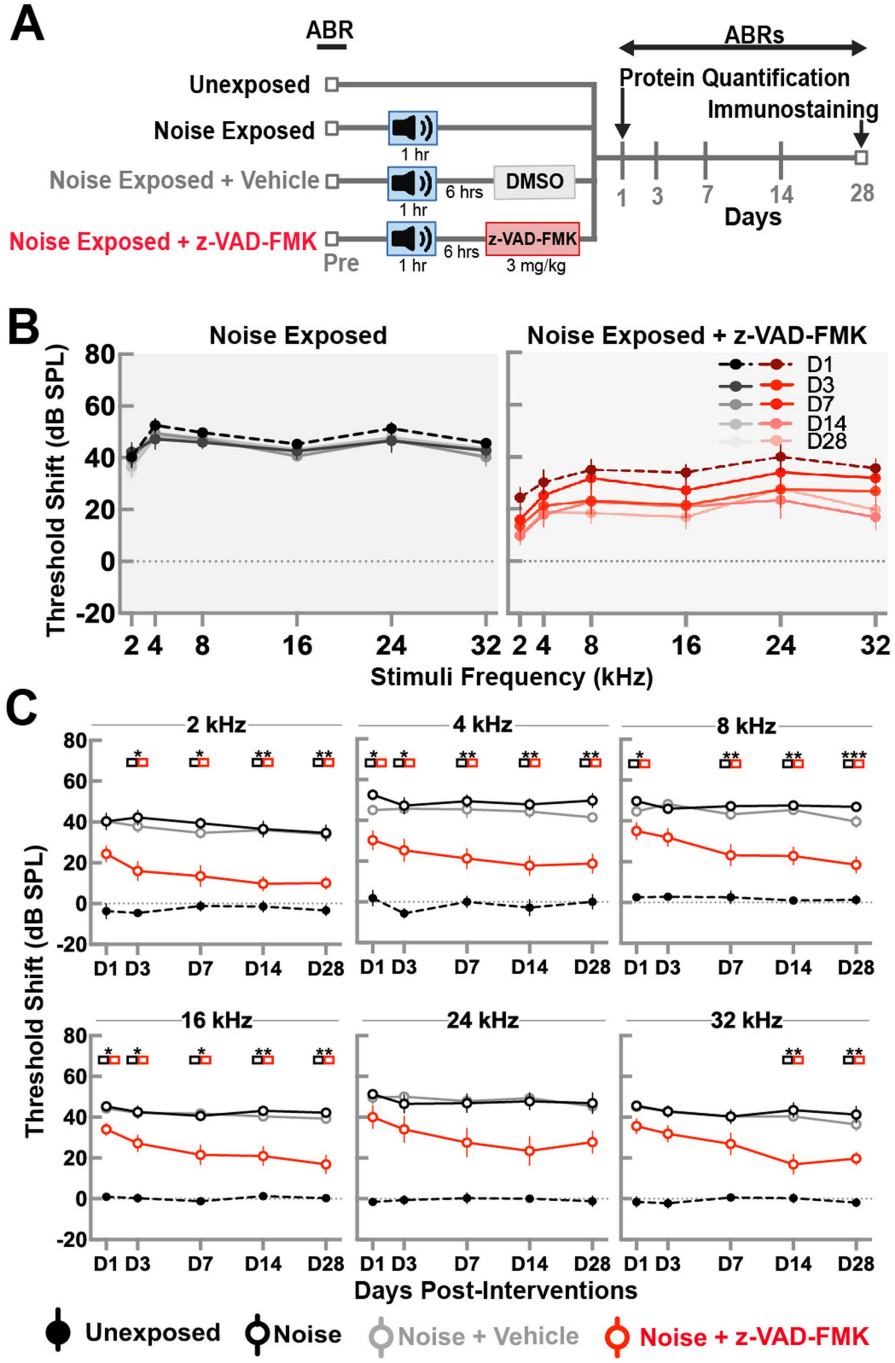
Post-noise administration of Z-VAD-FMK reduces ABR threshold shifts. **(A)** Schematic of the experimental setup. **(B)** ABR threshold shifts in the noise-exposed and noise + Z-VAD-FMK groups measured at 1, 3, 7, 14, and 28 days post-noise exposure for frequency stimuli of 2, 4, 8, 16, and 24 kHz. **(C)** ABR threshold shifts analyzed by individual frequencies across all groups: unexposed (*n* = 8, black dashed line), noise-exposed (*n* = 8, black), noise-exposed + vehicle (*n* = 8, gray), and noise + Z-VAD-FMK (*n* = 8, red). Data are presented as mean ± SE of ABR threshold shifts. Statistical significance between the noise-exposed and noise + Z-VAD-FMK groups at individual time points is denoted by colored boxes (^*^p < 0.05, ^**^p = 0.05–0.001, and ^***^p < 0.001).

To determine functional differences between groups, we performed auditory brainstem responses (ABRs) at five different time points following noise trauma. Figure 1A presents a schematic illustration of the experimental timeline that shows the four groups and time points for functional measurements, protein expression analysis and immunohistochemistry. Figure 1B shows the pattern of threshold shifts across all time points and frequencies for the noise-exposed and noise-exposed + Z-VAD-FMK-treated groups. Noise-exposed animals exhibited increased threshold shifts on day 1 at all frequencies which did not recover over the 28-day period, indicating a permanent threshold shift. The mean ± SE threshold shift across all frequencies in this group was 47.5 ± 2.7 dB on day 1 and 43.8 ± 3.8 dB on day 28. Animals treated with a single intraperitoneal (I.P.) injection of Z-VAD-FMK after noise exposure showed an initial increase in threshold shifts across all frequencies; however, these shifts gradually recovered over time. The mean ± SE threshold shift for the treated group across all frequencies was 33.5 ± 4.5 dB on day 1 and 18.6 ± 4.4 dB on day 28.

Figure 1C depicts ABR threshold shifts for all groups, stratified by individual frequencies. Data between groups were compared using two-way ANOVA with Tukey’s correction for multiple comparisons (^*^p < 0.05, ^**^p = 0.01–0.05, and ^***^p < 0.01).

Unexposed animals showed minimal threshold shifts across all frequencies, averaging −0.25 ± 2.6 dB on day 1 and −0.8 ± 2.5 dB on day 28. Significant differences were consistently observed between unexposed and noise-exposed groups, with no variations between noise-exposed and noise + vehicle groups.

Significant differences between noise-exposed and noise-exposed + Z-VAD-FMK groups were prominent at low (2 and 4 kHz) and mid (8 and 16 kHz) frequencies across most timepoints, with exceptions at day 3 post-intervention at 8 kHz. While a pattern of decreased shifts was evident, fewer significant results were observed in high frequencies, particularly at 24 kHz.

Sex-specific mean ± SE of amplitude and latency thresholds changes for all frequencies are presented in Supplementary Table S1.

### 3.2 Post-noise delivery of Z-VAD-FMK preserved ABR amplitudes and latencies

**Figure 2:**
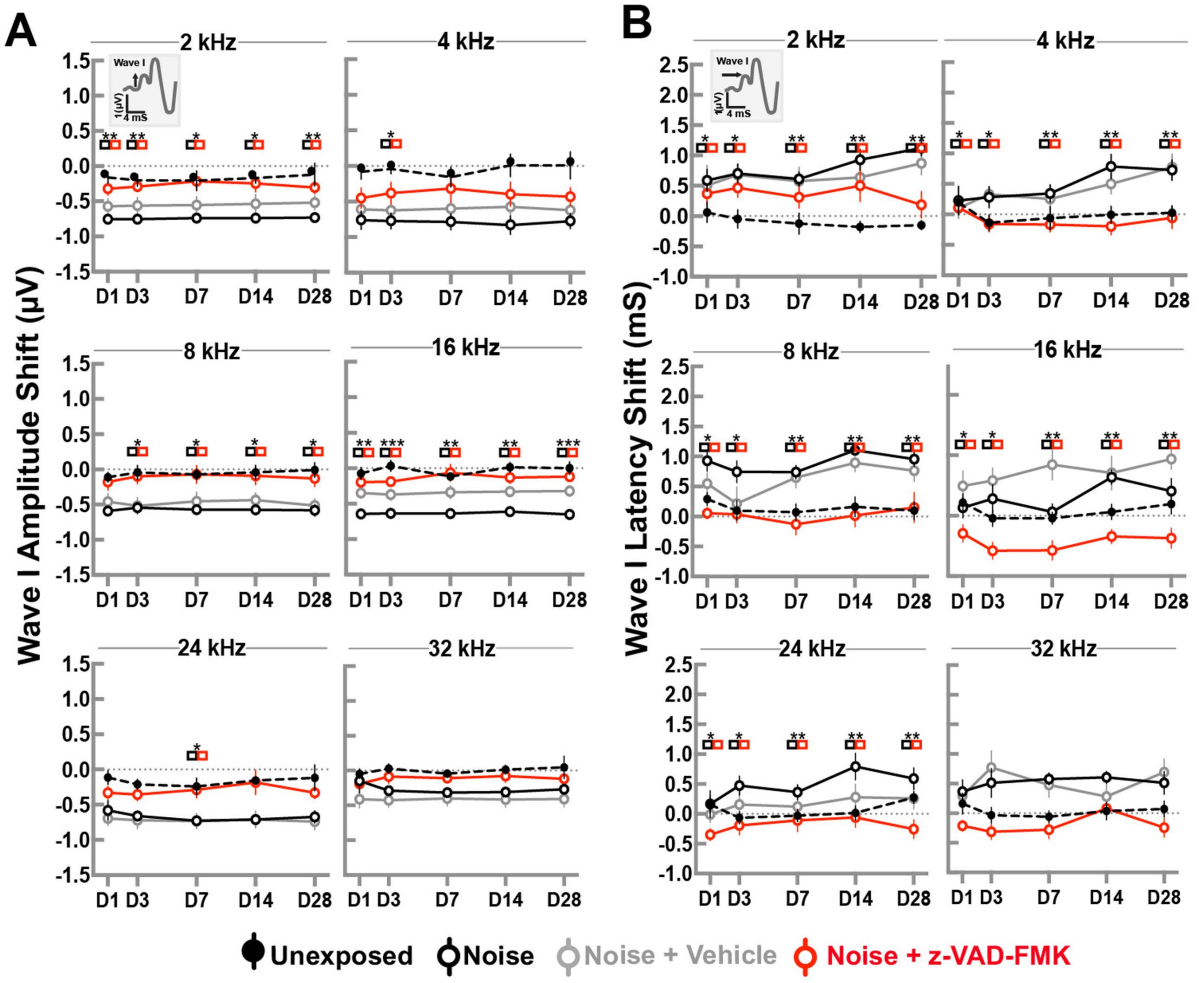
Z-VAD-FMK preserves ABR wave I amplitudes and latencies. **(A)** Shifts in wave I amplitudes and **(B)** shifts in wave I latencies at 80 dBA across all cochlear turn frequencies. Data are presented as mean ± SE. Statistical comparisons between the noise-only (black) and noise + Z-VAD-FMK (red) groups are indicated in colored boxes (^*^p < 0.05, ^**^p = 0.05–0.001, and ^***^p < 0.001).

ABR wave I peak amplitude changes at a suprathreshold level of 80 dBA were recorded to evaluate the effects of trauma and treatment on the peripheral cochlear nerve. Changes across all frequency stimuli are shown in Figure 2. Group comparisons at each time point were performed using two-way ANOVA with Tukey’s correction (^*^p < 0.05, ^**^p = 0.01–0.05, and ^***^p < 0.01).

Amplitude changes in the unexposed and noise-exposed groups showed minimal variations across frequencies and timepoints. Overall, changes averaged −0.08 ± 0.02 dB and −0.61 ± 0.02 dB, respectively. Significant differences were observed between these groups, but were not consistently found between noise-exposed and noise + vehicle groups. Animals treated with Z-VAD-FMK exhibited reduced changes compared to the noise-exposed group, with averages of −0.28 ± 0.04 dB, −0.39 ± 0.07 dB, −0.11 ± 0.05 dB, −0.14 ± 0.03 dB, −0.29 ± 0.05 dB, and −0.12 ± 0.03 dB at 2, 4, 8, 16, 24, and 32 kHz, respectively. Significant changes were predominantly observed at low and mid frequencies.

A similar pattern emerged in ABR latency changes (Figure 2B). Latency changes in unexposed and noise-exposed groups remained mostly consistent, averaging 0.04 ± 0.02 dB and 0.57 ± 0.03 dB, respectively, with no significant differences between noise + vehicle and noise-only groups.

Z-VAD-FMK-treated group showed notable differences from the noise-exposed group, particularly at low and mid frequencies (8 and 16 kHz), averaging −0.02 ± 0.07 dB and −0.43 ± 0.06 dB. High frequencies (24 and 32 kHz) showed averages of −0.19 ± 0.07 dB and −0.19 ± 0.06 dB, respectively.

Sex-specific mean ± SE of amplitude and latency changes for all frequencies are presented in Supplementary Table S2.

### 3.3 Pan-caspase inhibitor protects against noise-induced cochlear hair cell loss

**Figure 3:**
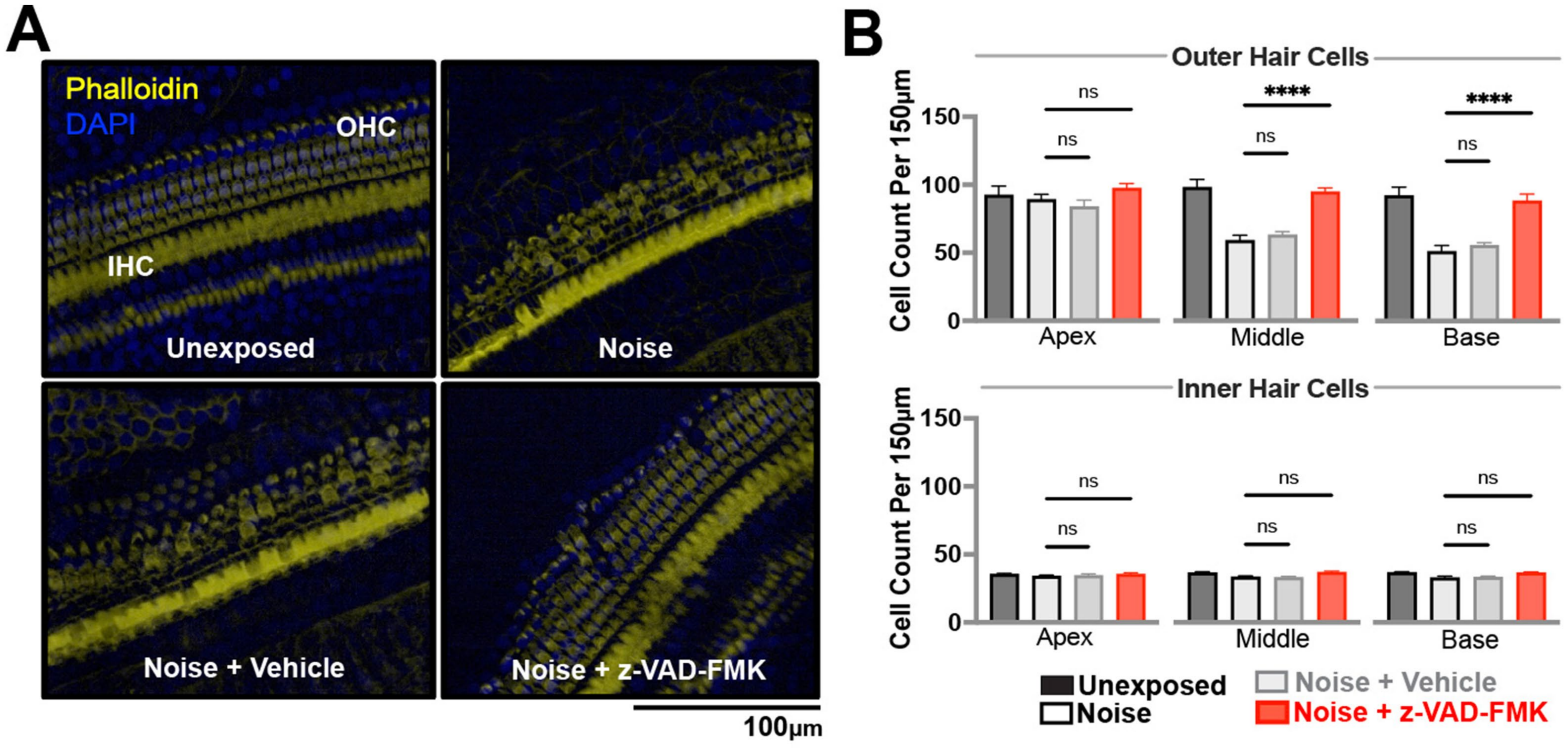
Z-VAD-FMK preserves cochlear hair cell density. **(A)** Representative confocal images of cochlear hair cells from the middle turn, stained with DAPI (blue) and phalloidin (yellow), captured at 20× magnification. Data were collected from unexposed, noise-exposed, noise + vehicle, and noise + Z-VAD-FMK groups at 28 days post-intervention. **(B)** Cochlear hair cell counts from the apical, middle, and basal turns, corresponding to low, mid, and high-frequency regions, respectively. Data are presented as mean ± SE. Statistical comparisons between the noise-exposed (black) and noise + Z-VAD-FMK (red) groups, as well as between the noise and noise + vehicle groups (gray), are shown (^*^p < 0.05, ^**^p = 0.05–0.001, and ^***^p < 0.001). Group sizes: unexposed (*n* = 8), noise (*n* = 8), noise + vehicle (*n* = 8), noise + Z-VAD-FMK (*n* = 8). IHC, inner hair cells; OHC, outer hair cells.

To investigate histological differences between non-treated and anti-apoptotic treatment groups, we assessed outer and inner hair cell survival across apical, middle, and basal cochlear turns. Phalloidin and DAPI staining allowed for quantification of cells (Figure 3A), with statistical analysis performed by two-way ANOVA and Tukey corrections (^*^p < 0.05, ^**^p = 0.01–0.05, and ^***^p < 0.01).

Unexposed group hair cell counts were consistent across turns, averaging 102 ± 0.8 outer hair cells and 35 ± 0.1 inner hair cells per 150 microns (Figure 3B). Noise-exposed animals showed decreased hair cell counts: outer hair cells averaged 65 ± 10.7 across turns while inner hair cells averaged 33 ± 0.6, with significant differences compared to unexposed groups. No significant variations were observed between noise-exposed and noise + vehicle groups.

Z-VAD-treated animals demonstrated increased hair cell survival, particularly in outer hair cells: 98 ± 3.1 (apical), 95 ± 2.8 (middle), and 88 ± 3.8 (basal) turns. Inner hair cell counts averaged 35 ± 0.9, 36 ± 0.9, and 36 ± 0.6 across turns.

### 3.4 Z-VAD-FMK attenuates noise-induced inflammatory and apoptotic protein responses

**Figure 4:**
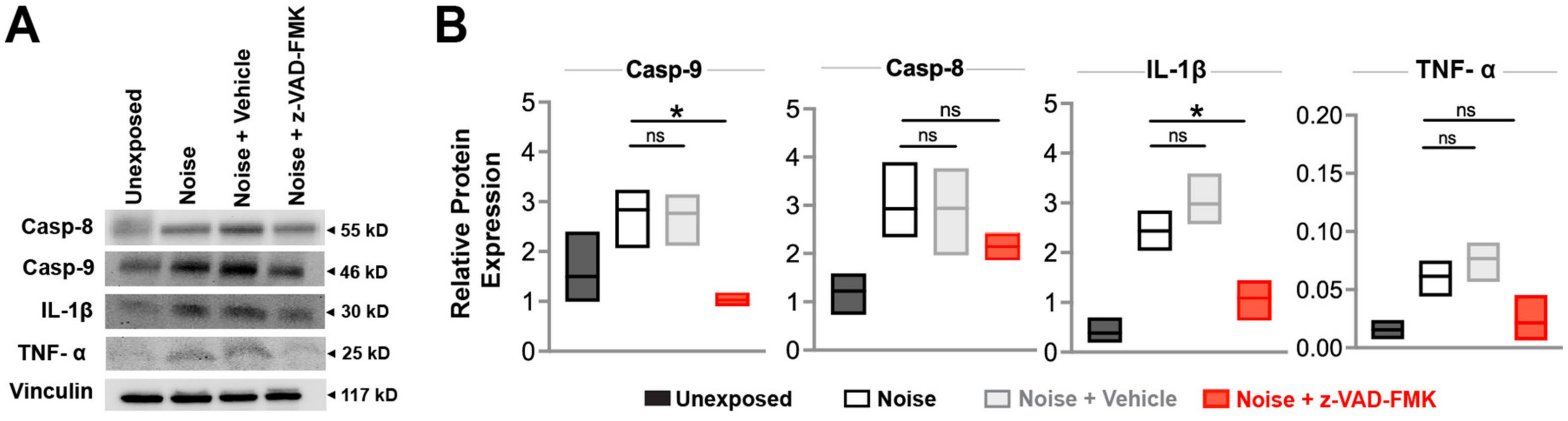
Z-VAD-FMK reduces noise-induced inflammatory and apoptotic protein levels. **(A)** Western blot analyses of inner ear protein levels of caspase-9, caspase-8, IL-1β, and TNF-α across all groups at 24 h post-intervention. Vinculin was used for normalization. **(B)** Relative protein expression levels for each group are presented as box plots showing the mean and upper and lower quartiles. Statistical comparisons between the noise-exposed (black) and noise + Z-VAD-FMK (red) groups, as well as between the noise-exposed and noise + vehicle groups (gray), are indicated (^*^p < 0.05, ^**^p = 0.05–0.001, and ^***^p < 0.001). Group sizes: unexposed (*n* = 3), noise (*n* = 3), noise + vehicle (*n* = 3), noise + Z-VAD-FMK (*n* = 3). Casp-9, caspase-9; Casp-8, caspase-8; IL-1β, interleukin-1β; TNF-α, tumor necrosis factor-α.

Western blot analyses were performed on male subjects 24 h post-intervention to quantify apoptotic and inflammatory proteins in the inner ear. Caspase-9, caspase-8 (apoptosis pathway indicators), IL-1β, and TNF-α (inflammation markers) were analyzed across unexposed, noise, noise-exposed + vehicle, and noise + Z-VAD-FMK groups (Figure 4A).

Relative protein expression of caspase-9 averaged 1.5 ± 0.4 in the unexposed group, 2.8 ± 0.3 in the noise-exposed group, 1.02 ± 0.06 in the noise + z-VAD-FMK, and 2.77 ± 0.3 in the noise +vehicle group. A significant reduction was observed between the noise-exposed and noise + Z-VAD groups while no significant differences were detected between the noise-exposed and noise + vehicle groups. IL-1β protein expression showed a similar pattern, with averages of 0.38 ± 0.1, 2.4 ± 0.2, 1.1 ± 0.2, and 2.9 ± 0.5 for the unexposed, noise-exposed, noise + z-VAD-FMK, and noise + vehicle groups, respectively, with significant differences noted between the noise-exposed and noise + Z-VAD groups (Figure 4B).

Caspase-8 and TNF-α levels followed a trend where treatment reduced protein expression, although these reductions were not statistically significant compared to the noise-exposed group. Specifically, caspase-8 protein expression averaged 2.5 ± 0.2 in the noise-exposed group and 2.1 ± 0.2 in the treated animals. TNF-α levels were 0.06 ± 0.1 in the noise-exposed group and 0.02 ± 0.01 in the noise + Z-VAD group (Figure 4B).

### 3.5 Treatment with Z-VAD-FMK is does not impact the unexposed auditory system

To assess the safety of a single injection of Z-VAD-FMK and the vehicle in non-exposed animals, two additional groups (*n* = 4 per group) were included in this study. The aim was to determine whether the drug and/or the vehicle alone had any inherent effects independent of trauma. Male Brown Norway rats (total *n* = 8) received the same dosing and timing as the other experimental groups. Supplementary Figure S1A presents auditory threshold shift data from both groups across all frequencies and time points, with shifts ranging between 10 and −10 dBA for all groups. Amplitude (Supplementary Figure S1B) and latency (Supplementary Figure S1C) shifts are shown for all frequencies, with shifts ranging between 0.5 and −0.5 μV and mS, respectively. Outer and inner hair cells at the apex, middle, and base of the cochlea were quantified following immunohistochemistry (Supplementary Figures S2A,B). Hair cell counts were not significantly different from those in the unexposed group. Similarly, caspase-9, caspase-8, IL-1β, and TNF-α protein levels did not differ from unexposed levels when quantified through western blotting (Supplementary Figures S2C,D).

## 4 Discussion

In this study, we explored the neuroprotective properties of Z-VAD-FMK, a pan-caspase inhibitor, in mitigating the functional, histological, and molecular damage caused by noise overexposure. Using validated assessments in a preclinical rodent model, we demonstrated that Z-VAD-FMK can partially reduce auditory dysfunction, preserve cochlear hair cells, and reduce levels of apoptotic and inflammatory pathway proteins in noise-exposed inner ears. Moreover, delivery of Z-VAD-FMK to naive animals did not impact any of the outcomes measured, suggesting its potential safety and lack of adverse effects on the auditory system independent of trauma.

### 4.1 Therapeutic strategies for cochlear damage

The management of noise-induced hearing loss (NIHL) today is primarily centered on preventive strategies and symptomatic treatment, as no current therapies can reverse cochlear damage once it occurs (Tikka et al., 2017; Neitzel and Seixas, 2005). Preventive measures, such as the use of hearing protection devices and strict regulation of noise exposure, are essential, particularly in occupational settings, as recommended by NIOSH guidelines (Sriwattanatamma and Breysse, 2000). Corticosteroids are commonly administered to reduce cochlear inflammation in cases of acute acoustic trauma, though their effectiveness in managing gradual hearing loss from continuous lower-level noise exposure remains debated (Zloczower et al., 2022; Chang et al., 2017; Choi et al., 2019; Takemura et al., 2004). In addition, despite their frequent use, corticosteroids are not universally considered the optimal standard of care due to potential side effects, such as high blood pressure (Koochakzadeh et al., 2020). This has led researchers to explore alternative treatments, including vascular agents, antioxidants (Pisani et al., 2023), and hyperbaric oxygen therapy (Lafère et al., 2010), although these approaches lack sufficient clinical evidence for widespread adoption in practice.

Z-VAD-FMK has emerged as a compelling therapeutic candidate for noise-induced hearing loss (NIHL) due to its targeted inhibition of apoptosis, the primary biological process leading to cell death following noise exposure (Hu et al., 2002; Hu et al., 2006). Additionally, it inhibits caspases 8 and 1, preventing inflammasome formation, pyroptotic pathways, and the subsequent inflammatory cascades that may exacerbate cochlear damage (Chauvier et al., 2007). These mechanisms suggest that Z-VAD-FMK could be more effective than other drugs targeting secondary processes, such as inflammation or oxidative stress. Notably, while it has demonstrated effectiveness in protecting hair cells from ototoxic drugs (Chang et al., 2020; Okuda et al., 2005) and oxidative stress (Singh et al., 2021), its potential to mitigate noise-induced damage remains unexplored.

### 4.2 Protective effects of Z-VAD-FMK

ABR analysis demonstrated a significant recovery of auditory thresholds mostly on the low and mid-frequencies following Z-VAD-FMK treatment. This recovery was evident as early as day 1 post-exposure and remained stable through day 28, indicating a sustained neuroprotective effect. ABR amplitudes and latencies, which represent the strength and timing of auditory nerve firing, respectively, were significantly improved with the anti-apoptotic treatment, particularly in the low to mid frequencies corresponding to apex and middle turns of the cochlea. Furthermore, histological analysis showed that Z-VAD-FMK significantly preserved outer hair cells, predominantly in the middle and basal turns of the cochlea, compared to the noise-only group.

Functional and histological evidence from this study highlights several key findings: (1) systemic administration of Z-VAD-FMK appears to have effectively reached multiple cochlear turns of the cochlea, offering significant advantages over more invasive methods, such as intratympanic or intracochlear injections, which primarily target the basal turn (Wang et al., 2018; Patel et al., 2019). This is particularly important given that broadband noise, both in this study and in real-world conditions, impacts a wide range of cochlear frequencies, necessitating treatments capable of reaching multiple regions. To ensure comprehensive protection, higher doses, repeated administrations, or combination therapies may be required. (2) Z-VAD-FMK not only successfully reached its target but also reduced outer hair cell (OHC) loss, especially in the middle and basal turns. Preservation of OHCs in the middle turn was correlated with improvements in auditory function at corresponding frequencies; however, despite hair cell preservation in the basal turn, functional gains in high-frequency regions, as measured by ABR, were limited. This suggests that while hair cell preservation is achieved, it does not fully restore function at these frequencies, potentially due to additional damage mechanisms, such as synaptic or neural deficits, which were not assessed in this study. Interestingly, in the apical turn, Z-VAD-FMK treatment improved functional measures without significantly affecting OHC counts, suggesting that, at the given dose and delivery method, Z-VAD-FMK may not fully address other damage mechanisms leading to hair cell death, though surviving hair cells in the apex appear capable of compensating to preserve function. Furthermore, inner hair cells (IHCs) across all cochlear turns remained largely unaffected in all groups, including those exposed to noise, consistent with the understanding that OHCs are more susceptible to less intense noise exposures. (3) Lastly, the absence of significant differences in functional outcomes and hair cell counts between the noise-exposed and noise + vehicle groups underscores the specificity of Z-VAD-FMK’s protective effects, as the vehicle alone did not provide substantial protection.

### 4.3 Underlying mechanisms of Z-VAD-FMK’s protective effects

Noise-induced cochlear damage is driven by several interrelated biological processes (Wang et al., 2018). A key mechanism involves the acceleration of cellular metabolism, resulting in the overproduction of free radicals and reactive oxygen species (ROS) by the mitochondria (Ohlemiller et al., 1999). This promotes the release of cytochrome c and initiates the apoptotic cascade, as evidenced by elevated levels of caspases 3, 8, and 9 in the inner ear of rodents exposed to noise (Nicotera et al., 2003). These caspase levels have been shown to rise shortly after acoustic trauma, peaking on day 1 with a secondary peak on days 7–8 (Wang et al., 2018). Furthermore, acoustic trauma triggers an immune response in the cochlea, characterized by the production of inflammatory mediators, such as interleukins and tumor necrosis factor, alongside the recruitment of immune cells (Wood and Zuo, 2017; Kalinec et al., 2017). This inflammatory response begins immediately post-trauma, with peaks observed between days 1–3 and 6–8 (Patel et al., 2019).

Based on the pathophysiological timeline, we conducted western blot analysis 24 h post-exposure to evaluate the intrinsic and extrinsic apoptosis pathways, targeting caspase-9 and caspase-8, respectively. Caspase-9 exhibited significant inhibition, while caspase-8 levels were reduced but not statistically significant. We also measured IL-1β and TNF-α, as they are key components of the inflammasome pathway activated by caspase-1 and are known to increase following noise exposure. IL-1β expression was significantly decreased with treatment, while TNF-α levels were reduced but not significantly. These results suggest several important points. First, although pharmacokinetic data for Z-VAD-FMK in the inner ear is limited, the observed reduction in protein levels indicates effective drug delivery to cochlear tissue (Shi, 2016). Second, the decrease in capase-9 and IL-1β levels suggests that Z-VAD-FMK is not only being effectively delivered but also successfully suppressing apoptosis and inflammation within cochlear hair cells. Third, the significant inhibition of caspase-9, alongside a non-significant reduction in caspase-8, supports the hypothesis that the intrinsic pathway is more heavily impacted in noise-induced hearing loss (NIHL), indicating that Z-VAD-FMK may be particularly effective in targeting this pathway. Fourth, timely administration of Z-VAD-FMK reduces both apoptotic and inflammatory responses during their peak activity, reinforcing the therapeutic window for the drug within the first few days after noise exposure, as established by the pathophysiological timeline. Finally, the lack of statistical significance in some markers, despite observed trends, suggests that Z-VAD-FMK may require higher doses or combination therapies for comprehensive protection.

## 5 Conclusions and limitations

The findings from this study provide evidence for the potential of Z-VAD-FMK as a therapeutic intervention for NIHL. Given the lack of FDA-approved treatments, Z-VAD-FMK represents a promising candidate for further development and clinical testing.

Future studies should explore the long-term effects of Z-VAD-FMK treatment, including its impact on hearing function beyond the 28-day post-noise exposure period. It will be important to determine the bioavailability of the compound within the inner ear, and monitor for any immediate or long-term adverse side effects associated with Z-VAD-FMK, both in terms of systemic health and auditory outcomes. Additionally, investigations into the optimal timing and dosage of Z-VAD-FMK administration could help refine its therapeutic application. Exploring combination therapies that include Z-VAD-FMK alongside other protective agents may enhance its efficacy and provide broader protection against the complex pathophysiology of NIHL. Further research should also examine synaptic integrity to assess the neuronal component of NIHL, offering a more complete understanding of how Z-VAD-FMK impacts both hair cells and the auditory nerve.

## Data Availability

The original contributions presented in the study are included in the article/Supplementary material, further inquiries can be directed to the corresponding author.
