# Supplementary material for: Pan-caspase inhibitor protects against noise-induced hearing loss in a rodent model
Source: Front Neurosci. 2025 Feb 10;19:1497773. doi: 10.3389/fnins.2025.1497773 (PMC11847858; doi:10.3389/fnins.2025.1497773)
Supplement: Supplementary file 1 [file Table_1.docx]

|  |  | **Threshold Shift (dBA)** | | | | | | | | | | | | | | | | | |
| --- | --- | --- | --- | --- | --- | --- | --- | --- | --- | --- | --- | --- | --- | --- | --- | --- | --- | --- | --- |
|  |  | **Stimuli Frequency** | | | | | | | | | | | | | | | | | |
|  |  | **2 kHz** | | | **4 kHz** | | | **8 kHz** | | | **16 kHz** | | | **24 kHz** | | | **32 kHz** | | |
|  | **DPI** | ***M*** | ***F*** |  | ***M*** | ***F*** |  | ***M*** | ***F*** |  | ***M*** | ***F*** |  | ***M*** | ***F*** |  | ***M*** | ***F*** |  |
| **Unexposed** | **1** | 4.5 (3.9) | -11.8 (3.8) | ns | 3.75 (7.5) | 0 (2.0) | ns | 5 (2.7) | 0 (3.1) | ns | -1.6 (1.3) | 3.1 (1.3) | ns | 0 (2.3) | -3.1 (4.7) | ns | -0.6 (3.3) | -2.5 (8.4) | ns |
|  | **3** | -2.4 (1.0) | -6.9 (4.2) | ns | -7.5 (4.2) | -3.8 (4.7) | ns | 3.8 (3.3) | 1.9 (4.7) | ns | 0.6 (3.1) | 0 (6.7) | ns | 0.6 (4.7) | -1.9 (1.3) | ns | 0 (4.7) | -4.4 (2.4) | ns |
|  | **7** | 1.4 (1.7) | -3.8 (9.2) | ns | -3.8 (5.2) | 3.8 (1.4) | ns | 1.9 (3.4) | 3.1 (11.4) | ns | -1.9 (0.6) | -0.6 (5.1) | ns | 3.8 (2.4) | -3.1 (8.3) | ns | 4.4 (1.2) | -3.1 (4.3) | ns |
|  | **14** | 0.1 (4.8) | -3.1 (5.9) | ns | -5 (8) | -0.6 (6.3) | ns | 2.5 (2.7) | -0.6 (5.2) | ns | 0.6 (1.2) | 1.0 (5.2) | ns | 1.3 (1.6) | -1.3 (4.8) | ns | 1.9 (4.1) | -1.3 (4.3) | ns |
|  | **28** | 0.1 (1.7) | -6.9 (8.9) | ns | -0.6 (6.8) | 0.63 (7.5) | ns | 0.6 (4.1) | 1.9 (5.5) | ns | -2.5 (1.8) | 3.1 (3.8) | ns | 2.5 (4) | -5 (5.4) | ns | 0 (3.7) | -3.8 (3.2) | ns |
| **Noise Exposed** | **1** | 40.6 (4.1) | 40 (15.5) | ns | 52.5 (1.0) | 52.5 (10.6) | ns | 49.4 (3.1) | 50 (2.9) | ns | 45 (2.0) | 45.6 (5.5) | ns | 54.5 (2.7) | 48.1 (6.6) | ns | 47.5 (1.4) | 43.8 (2.5) | ns |
|  | **3** | 40 (4.4) | 44.4 (12.8) | ns | 45.6 (2.6) | 48.8 (16.0) | ns | 45.6 (4.1) | 46.3 (5.9) | ns | 45 (3.5) | 40 (10.6) | ns | 50 (2.7) | 43.1 (17.3) | ns | 46.3 (2.4) | 39.4 (10.1) | ns |
|  | **7** | 39.4 (3.6) | 39.4 (8.0) | ns | 47.5 (1.4) | 51.3 (12.5) | ns | 47.5 (3.4) | 46.9 (6.3) | ns | 42.5 (1.4) | 38.8 (5.9) | ns | 51.3 (3.0) | 42.5 (16.9) | ns | 46.3 (2.6) | 34.4 (9.8) | ns |
|  | **14** | 40 (4.2) | 33.1 (13.8) | ns | 48.1 (3.3) | 47.5 (10.2) | ns | 49.4 (3.4) | 45.6 (6.9) | ns | 45 (2.5) | 41.3 (5.2) | ns | 53.1 (2.6) | 42.5 (15.1) | ns | 49.4 (2.1) | 37.5 (12.1) | ns |
|  | **28** | 36.9 (5.3) | 31.7 (11.2) | ns | 48.8 (3.3) | 50.6 (14.1) | ns | 46.9 (1.9) | 46.9 (7.2) | ns | 41.3 (1.6) | 43.1 (7.7) | ns | 52.5 (1.8) | 41.3 (19.6) | ns | 45.6 (2.8) | 36.9 (14.8) | ns |
| **Noise + z-VAD-FMK** | **1** | 23.1 (4.4) | 25.6 (14,1) | ns | 30 (3.7) | 30.6 (18.3) | ns | 36.3 (1.3) | 33.8 (17.6) | ns | 36.3 (0.7) | 31.9 (12.5) | * | 38.1 (5.7) | 41.9 (21.1) | ns | 35 (1.4) | 36.3 (15.6) | ns |
|  | **3** | 9.4 (6.7) | 22.5 (10.6) | ns | 21.9 (5.7) | 26.7 (11.5) | ns | 33.1 (1.2) | 30.6 (18.4) | ns | 31.3 (2.2) | 23.1 (15.9) | ns | 32.5 (6.8) | 35.6 (24.1) | ns | 31.9 (1.6) | 31.9 (17.1) | ns |
|  | **7** | 11.3 (9.4) | 15.6 (11.4) | ns | 19.4 (6.7) | 23.1 (16.3) | ns | 25.6 (6.3) | 20.6 (17.9) | ns | 26.9 (1.6) | 16.3 (18.3) | ns | 20.6 (7.1) | 31.9 (23.0) | ns | 29.4 (3.3) | 24.4 (21.6) | ns |
|  | **14** | 4.4 (4.8) | 15 (7.9) | ns | 17.5 (7.4) | 18.1 (13.9) | ns | 28.1 (3.7) | 17.5 (15.1) | ns | 28.1 (4.6) | 13.8 (13.1) | ns | 18.1 (8.1) | 26.5 (23.7) | ns | 16.6 (6.3) | 17.5 (16.7) | ns |
|  | **28** | 5.6 (4.3) | 9 (7.2) | ns | 18.8 (6.0) | 18.8 (16.5) | ns | 25.6 (3.4) | 11.6 (10.9) | ns | 23.1 (2.1) | 10.6 (16.6) | ns | 21.6 (5.8) | 31.9 (16.9) | ns | 18.1 (1.9) | 21.3 (12.7) | ns |
| **Noise + Vehicle** | **1** | 43.1 (2,4) | 36.7 (6.3) | ns | 42.5 (5) | 48.3 (2.9) | ns | 44.4 (5.9) | 45 (5.0) | ns | 42.5 (2.0) | 46.7 (5.8) | ns | 54.4 (8.0) | 43.3 (5.8) | ns | 45 (6.1) | 45 (5.0) | ns |
|  | **3** | 38.8 (4.3) | 36.7 (12.5) | ns | 43.8 (1.4) | 48.3 (11.5) | ns | 46.9 (4,2) | 50 (5.0) | ns | 40.6 (6.5) | 45 (0) | ns | 50 (7.9) | 50 (5.0) | ns | 42.5 (7.1) | 43.3 (5.8) | ns |
|  | **7** | 34.4 (5.5) | 35 (10) | ns | 40.6 (9.4) | 51.7 (10.4) | ns | 40.6 (8.5) | 46.7 (2.9) | ns | 40.6 (3.8) | 43.3 (2.9) | ns | 52.5 (6.4) | 41.7 (1.9) | ns | 40.6 (4.2) | 40 (8.7) | ns |
|  | **14** | 36.1 (2.4) | 35 (10) | ns | 42.5 (6.1) | 46.7 (11.5) | ns | 43.1 (3.8) | 48.3 (2.9) | ns | 40.6 (3.8) | 40 (5.0) | ns | 51.3 (7.5) | 46.7 (2.9) | ns | 40.6 (7.7) | 40 (5.0) | ns |
|  | **28** | 36.9 (2.4) | 30 (8.7) | ns | 40 (4.6) | 43.3 (2.8) | ns | 36.9 (5.2) | 43.3 (10.4) | ns | 37.5 (7.4) | 41.7 (5.8) | ns | 50.6 (7.2) | 38.3 (7.6) | ns | 37.5 (8.9) | 35 (8.7) | ns |

**Table 1. Summary of ABR Threshold Shifts**. Mean ± SE of threshold shifts from all groups separated by sex: Unexposed (n=4 males, n=4 females), Noise (n=4 males, n=4 females), Noise-Exposed + Vehicle (n=4 males, n=4 females), and Noise + z-VAD-FMK (n=4 males, n=4 females) groups. Data is shown for shifts at 2, 4, 8, 16, and 24 kHz stimuli across all time points (days 1, 3, 7, 14, and 28 post-intervention). Threshold shifts were determined by calculating the difference between post-exposure and pre-exposure values. The results of the statistical analyses comparing the average shifts between males and females at each time point and frequency are presented. DPI=Days post-interventions, M=Males, F=Females.
