# Supplementary material for: Pan-caspase inhibitor protects against noise-induced hearing loss in a rodent model
Source: Front Neurosci. 2025 Feb 10;19:1497773. doi: 10.3389/fnins.2025.1497773 (PMC11847858; doi:10.3389/fnins.2025.1497773)
Supplement: Supplementary file 2 [file Table_2.docx]

|  |  | **Amplitude Shift (uV)** | | | | | | | | | | | | | | | | | | |
| --- | --- | --- | --- | --- | --- | --- | --- | --- | --- | --- | --- | --- | --- | --- | --- | --- | --- | --- | --- | --- |
|  |  | **Stimuli Frequency** | | | | | | | | | | | | | | | | | | |
|  |  | **2 kHz** | | | **4 kHz** | | | | **8 kHz** | | | **16 kHz** | | | **24 kHz** | | **32 kHz** | | | |
|  | **DPI** | ***M*** | ***F*** |  | | ***M*** | ***F*** |  | ***M*** | ***F*** |  | ***M*** | ***F*** |  | ***M*** | ***F*** |  | ***M*** | ***F*** |  |
| **Unexposed** | **1** | -0.3 (0.1) | -0.04 (0.1) | ns | | -0.2 (0.1) | 0.04 (0.2) | ns | -0.1 (0.1) | -0.2 (0.03) | ns | 0.03 (0.1) | -0.2 (0.1) | ns | 0.1 (0.1) | -0.3 (0.1) | ns | 0.1 (0.1) | -0.1 (0.1) | ns |
|  | **3** | -0.21 (0.15) | -0.2 (0.1) | ns | | -0.1 (0.1) | -0.03 (0.1) | ns | 0.2 (0.1) | -0.3 (0.1) | ns | 0.1 (0.1) | -0.05 (0.1) | ns | -0.1 (0.1) | -0.3 (0.03) | ns | 0.1 (0.1) | -0.05 (0.02) | ns |
|  | **7** | -0.43 (0.2) | 0.01 (0.1) | ns | | -0.3 (0.2) | -0.02 (0.2) | ns | -0.03 (0.2) | -0.1 (0.03) | ns | -0.04 (0.04) | -0.2 (0.1) | ns | -0.2 (0.2) | -0.2 (0.2) | ns | -0.03 (0.1) | -0.005 (0.1) | ns |
|  | **14** | -0.2 (0.1) | -0.09 (0.05) | ns | | -0.1 (0.3) | 0.2 (0.2) | ns | -0.05 (0.2) | -0.04 (0.1) | ns | 0.1 (0.1) | -0.1 (0.1) | ns | -0.1 (0.1) | -0.2 (0.04) | ns | 0.03 (0.1) | 0.3 (0.2) | ns |
|  | **28** | -0.4 (0.2) | 0.7 (0.2) | ns | | -0.3 (0.2) | 0.3 (0.1) | ns | -0.1 (0.2) | 0.1 (0.1) | ns | -0.2 (0.01) | 0.2 (0.1) | ns | -0.3 (0.3) | 0.05 (0.2) | ns | -0.2 (0.2) | -0.004 (0.2) | ns |
| **Noise Exposed** | **1** | -0.7 (0.1) | -0.8 (0.1) | ns | | -0.7 (0.2) | -0.8 (0.2) | ns | -0.6 (0.2) | -0.6 (0.02) | ns | -0.6 (0.1) | -0.7 (0.1) | ns | -0.8 (0.1) | -0.4 (0.2) | ns | -0.5 (0.1) | -0.3 (0.02) | ns |
|  | **3** | -0.7 (0.1) | -0.8 (0.1) | ns | | -0.7 (0.2) | -0.9 (0.1) | ns | -0.5 (0.2) | -0.6 (0.04) | ns | -0.6 (0.1) | -0.7 (0.04) | ns | -0.7 (0.1) | -0.6 (0.1) | ns | -0.5 (0.03) | -0.3 (0.01) | ns |
|  | **7** | -0.7 (0.1) | -0.8 (0.1) | ns | | -0.7 (0.2) | -0.9 (0.2) | ns | -0.5 (0.2) | -0.6 (0.04) | ns | -0.6 (0.1) | -0.7 (0.05) | ns | -0.8 (0.1) | -0.7 (0.1) | ns | -0.6 (0.1) | -0.2 (0.1) | ns |
|  | **14** | -0.7 (0.1) | -0.8 (0.1) | ns | | -0.7 (0.2) | -0.9 (0.2) | ns | -0.5 (0.2) | -0.6 (0.03) | ns | -0.5 (0.1) | -0.7 (0.1) | ns | -0.7 (0.05) | -0.7 (0.1) | ns | -0.6 (0.1) | -0.23 (0.1) | ns |
|  | **28** | -0.7 (0.1) | -0.7 (0.1) | ns | | -0.7 (0.1) | -0.9 (0.2) | ns | -0.6 (0.2) | -0.6 (0.1) | ns | -0.6 (0.1) | -0.7 (0.1) | ns | -08 (0.1) | -0.6 (0.2) | ns | -0.5 (0.04) | -0.2 (0.1) | ns |
| **Noise +**  **z-VAD-FMK** | **1** | -0.5 (0.1) | -0.2 (0.1) | ns | | -0.6 (0.2) | -0.3 (0.1) | ns | -0.5 (0.2) | 0.02 (0.1) | ns | -0.07 (0.1) | -0.2 (0.1) | ns | -0.5 (0.1) | -0.2 (0.1) | ns | -0.2 (0.1) | -0.005 (0.2) | ns |
|  | **3** | -0.4 (0.1) | -0.2 (0.1) | ns | | -0.5 (0.3) | -0.3 (0.2) | ns | -0.4 (0.2_ | 0.1 (0.1) | ns | -0.1 (0.1) | -0.2 (0.1) | ns | -0.5 (0.1) | -0.2 (0.1) | ns | -0.2 (0.1) | -0.1 (0.1) | ns |
|  | **7** | -0.4 (0.2) | ;-0.03 (0.2) | ns | | -0.5 (0.3) | -0.1 (0.3) | ns | -0.3 (0.2) | 0.04 (0.2) | ns | -0.1 (0.1) | -0.01 (0.3) | ns | -0.4 (0.2) | -0.2 (0.2) | ns | -0.2 (0.1) | -0.1 (0.1) | ns |
|  | **14** | -0.4 (0.2) | 0.07 (0.1) | ns | | -0.6 (0.3) | -0.2 (0.2) | ns | -0.4 (0.2) | 0.1 (0.1) | ns | -0.1 (0.1) | -0.1 (0.2) | ns | -0.4 (0.2) | -0.09 (0.3) | ns | -0.03 (0.1) | -0.2 (0.02) | ns |
|  | **28** | -0.5 (0.2) | -0.2 (0.05) | ns | | -0.6 (0.2) | -0.3 (0.1) | ns | -0.5 (0.2) | 0.1 (0.1) | ns | -0.1 (0.1) | -0.2 (0.1) | ns | -0.4 (0.1) | -0.2 (0.1) | ns | -0.07 (0.1) | -0.3 (0.1) | ns |
| **Noise + Vehicle** | **1** | -0.7 (0.1) | -0.4 (0.1) | ns | | -0.7 (0.1) | -0.5 (0.1) | ns | -0.2 (0.6) | -0.3 (0.1) | ns | -0.5 (0.04) | -0.2 (0.1) | ns | -0.8 (0.2) | -0.6 (0.1) | ns | -0.5 (0.2) | -0.3 (0.03) | ns |
|  | **3** | -0.7 (0.1) | -0.3 (0.1) | ns | | -0.7 (0.2) | -0.51 (0.1) | ns | 0.2 (0.2) | -0.4 (0.04) | ns | -0.5 (0.1) | -0.2 (0.1) | ns | -0.8 (0.2) | -0.6 (0.03) | ns | -0.5 (0.2) | -0.3 (0.1) | ns |
|  | **7** | -0.7 (0.2) | -0.3 (0.1) | ns | | -0.7 (0.2) | -0.4 (0.1) | ns | 0.2 (0.2) | -0.3 (0.1) | ns | -0.5 (0.1) | -0.1 (0.1) | ns | -0.8 (0.2) | -0.6 (0.02) | ns | -0.5 (0.2) | -0.3 (0.01) | ns |
|  | **14** | -0.8 (0.1) | -0.2 (0.1) | ns | | -0.7 (0.2) | -0.5 (0.2) | ns | 0.2 (0.2) | -0.4 (0.03) | ns | -0.5 (0.1) | -0.2 (0.1) | ns | -0. (0.2) | -0.6 (0.01) | ns | -0.5 (0.2) | -0.3 (0.005) | ns |
|  | **28** | -0.7 (0.1) | -0.3 (0.1) | ns | | -0.7 (0.1) | -0.5 (0.1) | ns | 0.2 (0.2) | -0.4 (0.02) | ns | -0.5 (0.1) | -0.1 (0.1) | ns | -0.8 (0.2) | -0.6 (0.1) | ns | -0.5 (0.2) | -0.4 (0.1) | ns |
|  |  | **Latency Shifts (mS)** | | | | | | | | | | | | | | | | | | |
|  |  | **Stimuli Frequency** | | | | | | | | | | | | | | | | | | |
|  |  | **2 kHz** | | | **4 kHz** | | | | **8 kHz** | | | **16 kHz** | | | **24 kHz** | | **32 kHz** | | | |
|  | **DPI** | ***M*** | ***F*** |  | | ***M*** | ***F*** |  | ***M*** | ***F*** |  | ***M*** | ***F*** |  | ***M*** | ***F*** |  | ***M*** | ***F*** |  |
| **Unexposed** | **1** | 0.3 (0.1) | -0.2 (0.3) | ns | | 0.4 (0.1) | -0.04 (0.1) | ns | 0.3 (0.2) | 0.3 (0.2) | ns | 0.2 (0.3) | 0.2 (0.1) | ns | 0.1 (0.3) | 0.2 (0.1) | ns | 0.1 (0.3) | 0.3 (0.2) | ns |
|  | **3** | -0.01 (0.1) | -0.1 (0.3) | ns | | -0.1 (0.2) | -0.2 (0.2) | ns | 0.1 (0.2) | 0.1 (0.3) | ns | 0.1 (0.3) | -0.1 (0.1) | ns | -0.1 (0.2) | -0.1 (0.05) | ns | 0.1 (0.3) | -0.1 (0.1) | ns |
|  | **7** | -0.1 (0.1) | -0.1 (0.4) | ns | | -0.1 (0.2) | 0.02 (0.3) | ns | 0.05 (0.1) | 0.1 (0.3) | ns | 0.1 (0.1) | -0.2 (0.2) | ns | 0.01 (0.2) | -0.1 (0.1) | ns | 0.03 (0.1) | -0.1 (0.2) | ns |
|  | **14** | -0.1 (0.2) | -0.2 (0.1) | ns | | 0.1 (0.2) | -0.1 (0.1) | ns | 0.3 (0.3) | 0.01 (0.3) | ns | 0.2 (0.2) | -0.1 (0.1) | ns | 0.1 (0.2) | -0.1 (0.2) | ns | 0.3 (0.2) | -0.2 (0.2) | ns |
|  | **28** | -0.1 (0.1) | -0.2 (0.1) | ns | | 0.2 (0.2) | -0.1 (0.1) | ns | 0.3 (0.3) | -0.09 (0.1) | ns | 0.6 (0.1) | -0.2 (0.1) | ns | 0.5 (0.1) | 0.1 (0.1) | ns | 0.3 (0.2) | -0.2 (0.1) | ns |
| **Noise Exposed** | **1** | 0.6 (0.4) | 0.5 (0.2) | ns | | 0.3 (0.6) | 0.1 (0.2) | ns | 0.5 (0.3) | 0.9 (0.3) | ns | 0.9 (0.3) | 0.2 (0.3) | ns | 0.3 (0.2) | -0.1 (0.4) | ns | 0.9 (0.3) | 0.2 (0.2) | ns |
|  | **3** | 0.9 (0.3) | 0.5 (0.2) | ns | | 0.4 (0.4) | 0.1 (0.1) | ns | 0.5 (0.2) | 0.6 (0.3) | ns | 1.3 (0.1) | 0.1 (0.4) | ns | 0.1 (0.2) | 0.3 (0.2) | ns | 1.1 (0.5) | 0.3 (0.1) | ns |
|  | **7** | 0.7 (0.2) | 0.6 (0.1) | ns | | 0.3 (0.2) | 0.4 (0.2) | ns | 0.4 (0.3) | 0.9 (0.2) | ns | 0.9 (0.2) | 0.3 (0.2) | ns | 0.04 (0.2) | 0.3 (0.2) | ns | 0.9 (0.2) | 0.6 (0.2) | ns |
|  | **14** | 1.0 (0.2) | 0.8 (0.3) | ns | | 0.9 (0.4) | 0.9 (0.2) | ns | 0.7 (0.2) | 1.1 (0.1) | ns | 1.5 (0.2) | 0.5 (0.2) | ns | 0.9 (0.3) | 0.6 (0.3) | ns | 1.1 (0.2) | 0.6 (0.1) | ns |
|  | **28** | 1.1 (0.2) | 1.1 (0.2) | ns | | 0.7 (0.3) | 0.7 (0.1) | ns | 0.2 (0.3) | 0.9 (0.5) | ns | 0.9 (0.3) | 0.7 (0.2) | ns | 0.5 (0.3) | 0.6 (0.3) | ns | 0.6 (0.4) | 0.7 (0.3) | ns |
| **Noise +**  **z-VAD-FMK** | **1** | 0.6 (0.1) | 0.2 (0.3) | ns | | 0.2 (0.2) | -0.09 (0.1) | ns | 0.1 (0.3) | -0.01 (0.2) | ns | -0.1 (0.2) | -0.5 (0.2) | ns | -0.2 (0.2) | -0.5 (0.1) | ns | -0.1 (0.1) | -0.3 (0.1) | ns |
|  | **3** | 0.5 (0.2) | 0.4 (0.3) | ns | | 0.04 (0.2) | -0.4 (0.1) | ns | 0.1 (0.1) | 0.02 (0.3) | ns | -0.5 (0.2) | -0.7 (0.2) | ns | -0.4 (0.2) | -0.03 (0.3) | ns | -0.4 (0.1) | -0.2 (0.2) | ns |
|  | **7** | 0.4 (0.3) | 0.2 (0.3) | ns | | 0.09 (0.2) | -0.2 (0.2) | ns | -0.1 (0.2) | -0.2 (0.3) | ns | -0.3 (0.1) | -0.9 (0.2) | ns | -0.05 (0.3) | -0.2 (0.3) | ns | -0.1 (0.2) | -0.4 (0.2) | ns |
|  | **14** | 0.4 (0.1) | 0.6 (0.5) | ns | | -0.1 (0.2) | -0.3 (0.3) | ns | 0.03 (0.2) | -0.03 (0.3) | ns | -0.4 (0.2) | -0.3 (0.2) | ns | -0.3 (0.2) | 0.2 (0.2) | ns | 0.1 (0.2) | 0.1 (0.1) | ns |
|  | **28** | 0.1 (0.3) | 0.3 (0.4) | ns | | -0.2 (0.2) | 0.04 (0.3) | ns | 0.6 (0.2) | 0.1 (0.5) | ns | -0.3 (0.1) | -0.4 (0.3) | ns | -0.1 (0.2) | -0.4 (0.3) | ns | -0.3 (0.3) | -0.2 (0.2) | ns |
| **Noise + Vehicle** | **1** | 0.1 (0.5) | -0.3 (0.5) | ns | | 0.4 (0.3) | 0.3 (0.1) | ns | 0.6 (0.3) | 0.5 (0.3) | ns | 0.7 (0.2) | 0.3 (0.6) | ns | 0.4 (0.2) | 0.2 (0.1) | ns | 0.3 (0.2) | 0.1 (0.3) | ns |
|  | **3** | 0.4 (0.3) | 0.4 (0.3) | ns | | 0.09 (0.2) | 0.5 (0.1) | ns | 0.4 (0.4) | 0.3 (0.5) | ns | 0.7 (0.2) | 0.2 (0.4) | ns | -0.2 (0.2) | 0.8 (0.3) | ns | 0.7 (0.3) | 0.5 (0.1) | ns |
|  | **7** | 0.5 (0.3) | -0.02 (0.6) | ns | | 0.1 (0.3) | 0.5 (0.1) | ns | 0.4 (0.3) | 0.7 (0.1) | ns | 0.9 (0.2) | 0.4 (0.4) | ns | -0.09 (0.3) | 0.3 (0.1) | ns | 0.8 (0.3) | 0.3 (0.3) | ns |
|  | **14** | 0.5 (0.4) | 0.6 (0.5) | ns | | 0.3 (0.3) | 0.5 (0.01) | ns | 0.8 (0.2) | 0.8 (0.2) | ns | 1.1 (0.3) | 0.4 (0.5) | ns | 0.1 (0.3) | 0.4 (0.5) | ns | 0.6 (0.2) | -0.2 (0.3) | ns |
|  | **28** | 0.8 (0.4) | 0.7 (0.5) | ns | | 0.5 (0.3) | 0.6 (0.4) | ns | 0.9 (0.3) | 0.7 (0.1) | ns | 0.9 (0.3) | 0.8 (0.3) | ns | 0.2 (0.1) | 0.3 (0.4) | ns | 0.8 (0.3) | 0.3 (0.2) | ns |

**Table 2. Summary of ABR Latency and Amplitude Shifts.** Mean ± SE of amplitude and latency shifts from all groups separated by sex : Unexposed (n=4 males, n=4 females), Noise (n=4 males, n=4 females), Noise-Exposed + Vehicle (n=4 males, n=4 females), and Noise + z-VAD-FMK (n=4 males, n=4 females) groups. Data is provided for each frequency stimulus tested and all time points. Shifts were calculated as the difference between post-exposure and pre-exposure values. The results of the statistical analyses comparing the average shifts between males and females at each time point and frequency are presented. DPI=Days post-interventions, M=Males, F=Females.
