# Supplementary material for: Pan-caspase inhibitor protects against noise-induced hearing loss in a rodent model
Source: Front Neurosci. 2025 Feb 10;19:1497773. doi: 10.3389/fnins.2025.1497773 (PMC11847858; doi:10.3389/fnins.2025.1497773)
Supplement: Supplementary file 3 [file Data_Sheet_1.docx]

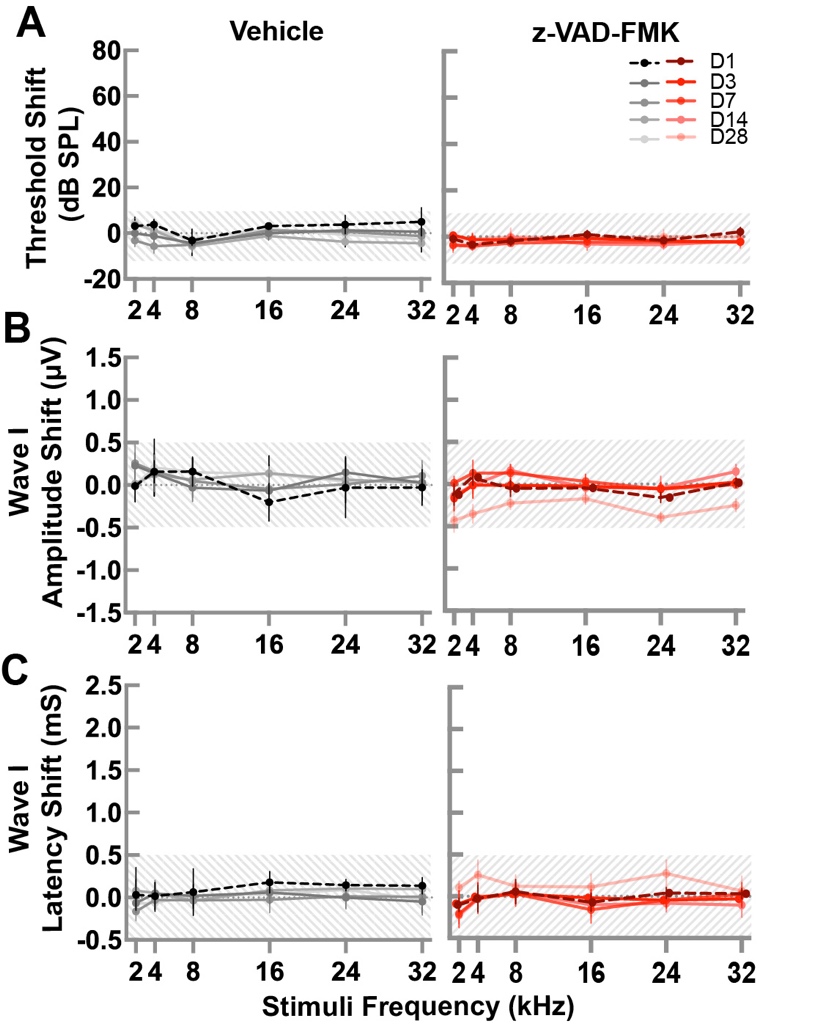


**Figure 1. Delivery of z-VAD-FMK and Vehicle Does Not Impact ABR Outcomes.** ABR threshold shifts (A), amplitude shifts (B) and latency shifts (C) data from the vehicle-only (n=4, gray lines) and z-VAD-FMK-only (n=4, red lines) groups measured at 1, 3-, 7-, 14-, and 28-days post-exposure for frequency stimuli at 2, 4, 8, 16, and 24 kHz. Data is presented as mean ± SE. The shaded gray area represents the range within which all recorded outcomes were recorded.
