# Supplementary material for: Pan-caspase inhibitor protects against noise-induced hearing loss in a rodent model
Source: Front Neurosci. 2025 Feb 10;19:1497773. doi: 10.3389/fnins.2025.1497773 (PMC11847858; doi:10.3389/fnins.2025.1497773)
Supplement: Supplementary file 4 [file Data_Sheet_2.docx]

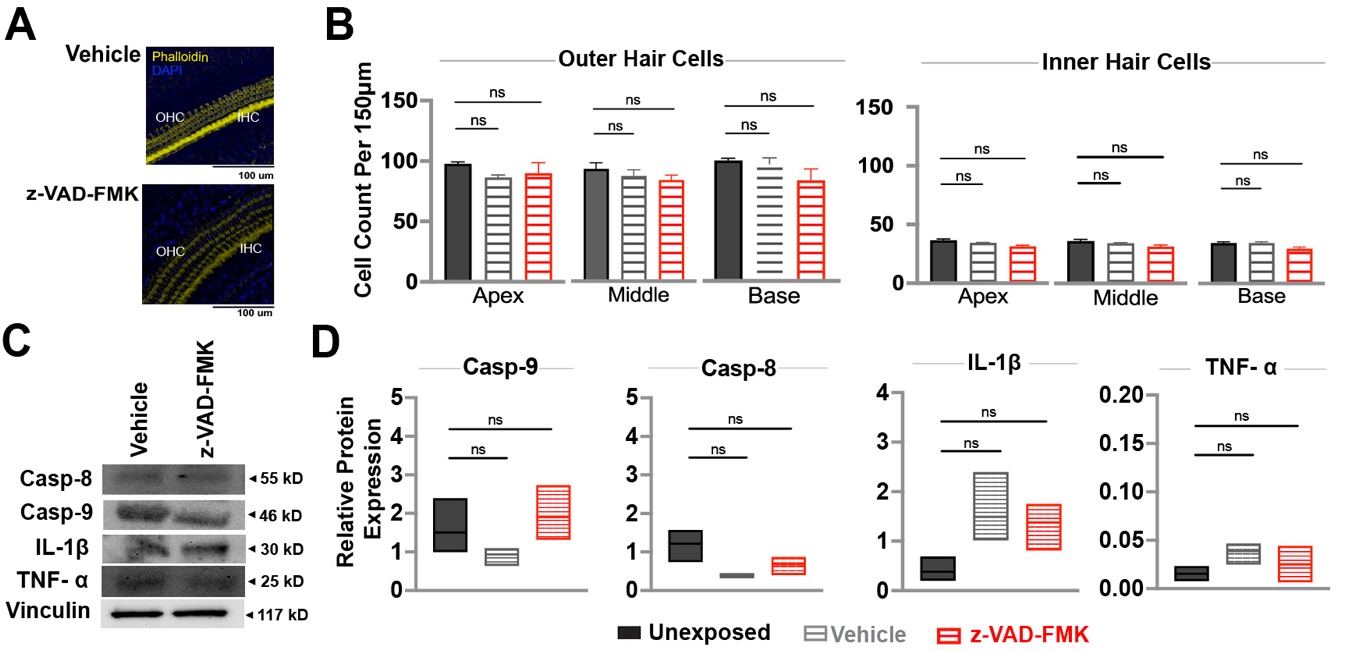


**Figure 2. Administration of z-VAD-FMK and the vehicle does not affect hair cell density or protein levels.** (A) Representative confocal images of middle turn cochlear hair cells stained with DAPI (blue) and Phalloidin (yellow) captured at 20x magnification. Data recovered at 28 post interventions is shown for the vehicle-only (n=4) and z-VAD-FMK-only (n=4) groups. (B) Quantified cochlear hair cells per 150um area from the apical, middle, and basal turns across all groups. (C) Representative protein band images from western blot analyses conducted on all groups(n=3/group). (D) Relative protein expression levels for each group presented as median and lower and upper quartiles. Statistical comparisons between the noise-exposed (black) and the noise + z-VAD-FMK (red) groups, as well as between the noise and noise + vehicle (gray) groups are shown. IHC=inner hair cells; OHC= outer hair cells; Casp-9= caspase-9; Casp8= caspas-8; IL-1B= interleukin-1B, TNF-alfa= tumor necrosis factor-alfa.
